# A Chronocosmetic Approach to Treating Signs of Aging with Glutathione

**DOI:** 10.3390/life15101623

**Published:** 2025-10-17

**Authors:** Candelaria Contreras-Agudelo, Amin Ariza-Donado, Mariana Villegas-Gallego, Aura Villa-Sierra, Jennifer Naranjo-Narvaez, Isabella Martinez-Meza

**Affiliations:** Dr. Amin Ariza Research and Innovation Center, Dr. Amin Ariza School, Barranquilla 080020, Colombia; direccion@escuelaaminariza.edu.co (A.A.-D.); aura.v@escuelaaminariza.edu.co (A.V.-S.); jenifer.n@escuelaaminariza.edu.co (J.N.-N.); isabella.m@escuelaaminariza.edu.co (I.M.-M.)

**Keywords:** cutaneous aging, oxidative stress, glutathione, anti-aging therapy, chronocosmetics, microneedling

## Abstract

Cutaneous aging is associated with oxidative stress and visible changes on the skin, such as uneven pigmentation, wrinkles, and loss of radiance. Glutathione (GSH), an endogenous antioxidant with anti-melanogenic properties, is considered a promising active agent in anti-aging protocols. This study evaluated a chronocosmetic approach based on the combined application of topical glutathione with microneedling, oral supplementation (250 mg), and a day-and-night home care routine. An 8-week interventional study was conducted with seven participants aged 30 to 60 years. Glutathione was applied in clinic using nanoneedles, along with a daily home care routine and oral administration. The progression of aging signs was assessed using 3D imaging, clinical photography, and cosmetic dermatology evaluation. The results showed improvements in pigmentation, radiance, and expression lines, with good tolerability, even in Fitzpatrick skin types III–IV.

## 1. Introduction

Among human organs, the skin is the one that most clearly reflects the signs of aging. It has a highly intricate microanatomical structure and fulfills several essential physiological roles. The pathophysiology of cutaneous aging is marked by the degradation of structural stability and functional integrity, leading to a progressive decrease in optimal performance and reserve capacity as a consequence of cumulative damage caused by intrinsic and extrinsic factors [1].

Cutaneous aging is characterized by structural and functional changes that lead to visible facial alterations. It is also associated with increased generation of reactive oxygen species (ROS) [2,3]; aesthetically, this results in fine lines, wrinkles, loss of elasticity and volume, rough texture, and uneven pigmentation. In the field of advanced cosmetology, the aim is to optimize anti-aging treatments by incorporating antioxidant agents such as glutathione, along with approaches based on chronotherapy to enhance efficacy. Glutathione is an endogenous tripeptide antioxidant essential to cellular defense against oxidative stress, and it also acts as a modulator of melanogenesis. Glutathione is a low-molecular-weight tripeptide ubiquitously present in eukaryotic cells, composed of three amino acids: L-Glutamic acid, L-Cysteine, and glycine. It plays a critical role in redox homeostasis and cellular detoxification processes, acting as a primary intracellular antioxidant [2,4].

Skin aging results from a complex interplay of intrinsic factors, such as telomere attrition, oxidative damage, hormonal changes, and reduced autophagic activity, and extrinsic factors, including exposure to ultraviolet light, environmental pollution, tobacco use, and dietary habits [5].

Recent studies have suggested that oral administration of glutathione (250–500 mg/day) can lighten the skin and improve parameters such as wrinkles and elasticity [4]. Moreover, chronocosmetics proposes synchronizing the application of cosmetic products with the skin’s circadian rhythms [6]. This study implements a combined protocol of weekly topical glutathione via microneedling, a daily home care routine, and oral supplementation (250 mg), aiming to objectively assess its impact on facial aging signs through 3D analysis.

Facial hyperpigmentation is an aesthetic concern that affects many individuals, impacting their self-esteem and quality of life. Glutathione, recognized for its antioxidant and anti-melanogenic properties, has emerged as a promising agent to reduce melanin synthesis by inhibiting tyrosinase and modulating the balance between eumelanin and pheomelanin. Furthermore, recent research suggests that synchronizing skin treatments with circadian rhythms (chronobiology) and optimizing application timing based on diurnal variations in skin function (chronocosmetics) can enhance the effectiveness of depigmenting therapies [2,7].

It has been demonstrated that the use of topical antioxidants helps protect the skin against harmful free radicals, whether generated intrinsically by normal cellular metabolism or induced by ultraviolet (UV) light exposure. Reactive oxygen species (ROS) are produced when biological macromolecules, such as lipids, proteins, and nucleic acids, interact with UV radiation. The main mechanism involves the direct absorption of UV radiation, which can excite and ionize molecules, thereby promoting the formation of free radicals [8].

### Theoretical Framework

Oxidative stress, primarily caused by external factors such as sleep disturbances, sun exposure, emotional stress, and skin inflammation, leads to melanogenesis. Elevated serum markers of oxidative stress have been observed in patients with melasma, with a significant correlation between plasma glutathione peroxidase levels and melasma severity [9,10].

In a non-randomized study involving 30 women in Egypt, one group (*n* = 10) received 500 mg/day of oral glutathione, another used 2% topical glutathione, and a third group received placebo for four weeks, all of which were combined with SPF 30 sunscreen. Superior performance was observed in both the oral and topical groups, with no adverse effects reported. These results reinforce the role of antioxidants in the treatment of melasma [11].

Glutathione has also been shown to be effective in treating photoaging when used in combination protocols with other compounds. Although the use of antioxidant enzymes in cosmetic and dermatological products is well documented, their topical application is limited by unfavorable physicochemical properties. Therefore, an effective alternative is the use of cell-penetrating peptides (CPPs), which facilitate the transduction of macromolecules, including enzymes, across the skin barrier. A recent study employed a combination of the glutathione peroxidase enzyme from the fungus Trichoderma reesei (TrGPx) and CPPs as an active antioxidant compound for skin treatment [2].

There are also treatments for facial aging that combine ultrasound technology with topical components containing glutathione. The existing evidence indicates that ultrasound exhibits enhanced effects when used simultaneously with glutathione, significantly improving skin quality and luminosity without producing adverse effects [12].

Another study demonstrates the effectiveness of glutathione in combination with hyaluronic acid and other topical substances. These agents produce aesthetic improvement in patients with skin imperfections when administered via mesotherapy. The treatment lasted for 3 months and focused on wrinkles on the face, neck, décolleté, and hands. Favorable results were observed, including a reduction in wrinkles, along with improvements in skin texture, luminosity, and turgor—all achieved without adverse effects [13].

Glutathione is also used in dental aesthetics with very positive results. This is demonstrated by a case series whose primary objective was to evaluate the clinical outcomes of mesotherapy using a combination of vitamin C, tranexamic acid, and glutathione to treat melanin-induced gingival pigmentation. This condition is considered an aesthetic concern that affects patients’ ability to smile confidently. The treatment is recognized for involving minimally invasive procedures, and no side effects were reported [14].

Aiming to search for the most effective method for glutathione application, a recent study found that glutathione may be more effective when formulated as a hydrogel. Considering its inherent instability and limited penetration through the stratum corneum, topical application poses challenges. The hydrogel formulation of glutathione demonstrated desirable properties, including gel stability, spreadability, pseudoplastic behavior, and elasticity [15].

Most protocols are aimed at treating already-aged skin; however, information is also available on protocols for preventing premature skin aging. A literature review concludes that to enhance the management of skin aging and promote overall skin health, a holistic and multi-level approach that combines the external use of anti-aging topical formulations with internal oral supplementation is suggested. Furthermore, a range of natural-origin ingredients with potential anti-aging benefits has been evaluated. Many of these components demonstrate multiple bioactive properties and may play a significant role in creating the ideal anti-aging solution [1].

In recent studies, 3D cameras have been used to evaluate the effects of anti-aging protocols. An example of this was the assessment of the safety of exosomes derived from *Solanum tuberosum* and their anti-aging properties in medical and cosmetic treatments. This research demonstrated an improvement in clinical parameters, such as wrinkle reduction, enhanced elasticity, and skin whitening [16].

## 2. Materials and Methods

A cohort study was conducted with a purposive sample of 7 participants. The primary objective of the research was to evaluate the effectiveness of a combined glutathione protocol (topical intervention with microneedling + oral supplementation 250 mg) on signs of facial aging, implemented within a chronocosmetic framework.

The inclusion criteria were as follows: (a) individuals aged 30 to 60 years, (b) cosmetic evaluation indicating facial hyperpigmentation (lentigines, melasma, or solar macules), (c) no invasive aesthetic treatments in the past 6 months, and (d) willingness to comply with both in-clinic and home-based protocols.

The following were the exclusion criteria:

**General medical conditions**: Pregnancy or breastfeeding; uncontrolled systemic diseases (e.g., decompensated diabetes, uncontrolled hypertension, cardiovascular diseases, active autoimmune disorders); significant hepatic or renal impairment; immunosuppression; active cancer; bleeding disorders or treatment with anticoagulants/antiplatelet agents; known allergy/hypersensitivity to glutathione or to excipients in the serum/supplement.

**Dermatological conditions**: Active dermatoses; tendency toward pathological scarring; photosensitivity; moderate-to-severe inflammatory acne.

**Recent interventions or interfering drugs**: Oral isotretinoin within the past 6–12 months; invasive facial aesthetic procedures within the past 6 months (laser, medium/deep chemical peels, radiofrequency, HIFU, or injectables); current use of potent depigmenting agents or topical retinoids without an appropriate washout period (e.g., 4 weeks); photosensitizing drugs that cannot be discontinued or adjusted.

**Exposure and behavior**: Intense sun exposure or use of tanning beds during the study or within the previous 4–6 weeks, when not controllable; anticipated lack of adherence to clinic visits or home routine (as judged by the investigator); problematic alcohol or substance use compromising adherence/safety.

**Methodological/ethical aspects**: Age outside the inclusion range (30–60 years); lack of informed consent or inability to understand protocol instructions.

**Figure 1:**
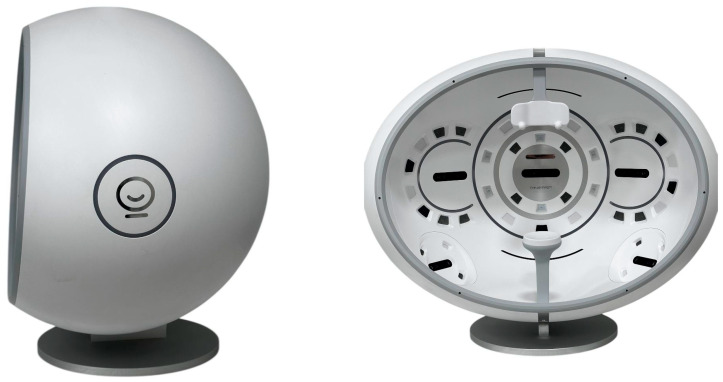
Three-dimensional camera used in this study.

The initial phase included an analytical and photographic baseline assessment (using a 3D camera (Kingtronics Corporation of Elec. & Mech. Technology (Zhangzhou) Co., Ltd., Zhangzhou, China) (Figure 1), skin evaluation under Wood’s lamp, and the application of a skin preparation protocol.

Epidermal hyperpigmentation was observed in all participants, which predicted a better response to topical treatments since the alteration was located in the superficial layer of the skin. Signs of aging were also evident, such as expression lines, enlarged pores, and skin laxity.

**Units of measurement**:

**Proportion**: Percentage of the facial skin presenting hyperpigmentation.

**Chloasma**: Levels (1–4), where 1 = mild and 4 = severe.

**Pigmented spots**: Levels (1–4), where 1 = mild alteration and 4 = severe alteration.

**Freckles**: Count of the total number of freckles in the area analyzed by the camera.

**Pigmentation**: Score (1–100), where 1 = mild, 50 = moderate, and 100 = severe.

**Crow’s feet**: Range (1–8), where 1 = mild, 4 = moderate, and 8 = severe.

**Pores**: Fine or coarse, with values ranging from 1 to 100, where 1 = fine, 50 = moderate, and 100 = coarse. Expected value: Fine.

## 3. Results

### Statistical Analysis

All variables were analyzed across three repeated measurements (baseline, session 4, and session 7). Pigmentation proportion was expressed as a dimensionless ratio, whereas chloasma, freckles, and pigmentation spots were reported as absolute lesion counts. Dynamic wrinkles (crow’s feet) were quantified on an ordinal scale reflecting severity, while pore condition was expressed as a numeric score.

Descriptive statistics showed that at baseline, the mean pigmentation proportion was 0.15 ± 0.10, with an average of 9.9 ± 4.1 chloasma lesions and 396 ± 215 freckles. The average number of pigmentation spots was 43 ± 27, and the severity of crow’s feet scored 4.3 ± 1.5 points. Pore condition presented a mean score of 57 ± 18.

When comparing between the sessions, a reduction in pigmentation proportion and freckle counts was observed over time, accompanied by a modest improvement in crow’s feet scores and pore condition. Inferential testing using repeated-measures ANOVA confirmed a statistically significant change across sessions for pigmentation proportion (*p* < 0.05) and freckle counts (*p* < 0.01), while changes in chloasma and pore scores did not reach statistical significance (*p* > 0.05).

Overall, the longitudinal analysis supports that skin condition improved progressively during the intervention period, particularly regarding pigment-related parameters, whereas wrinkle and pore metrics showed more variable trends. The overall progress achieved by the camera for each participant will be described below. If you need to provide an example of the camera images for each indicator, you can review the supplementary material.

**Figure 2:**
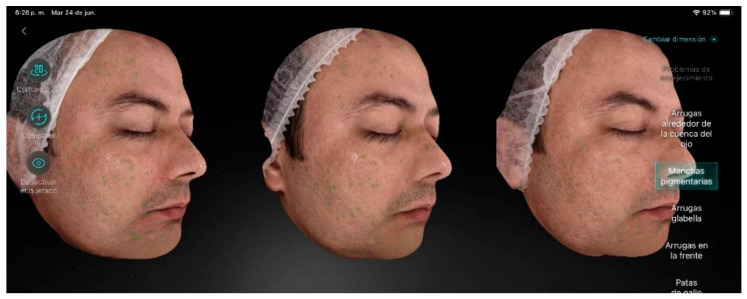
Participant 1 showing reduction in pigmented spots, freckles, and periorbital wrinkles (crow’s feet), along with improvement in radiance and pores.

Participant 1 (Figure 2), aged 44, has a history of thyroid disease. The analytical evaluation revealed combination skin, a low level of aging, and visibly rough skin texture with some fine lines. The main factor contributing to aging was hyperpigmentation, particularly on the cheekbones, and enlarged pores were also noted. The melasma observed under Wood’s lamp was located in the epidermis. The results from the last treatment session, as quantified using the 3D camera and verified by the cosmetologist, showed a reduction in pigmented spots, number of freckles, and periorbital wrinkles, as well as a decrease in pore size and an improvement in skin texture and brightness.

**Figure 3:**
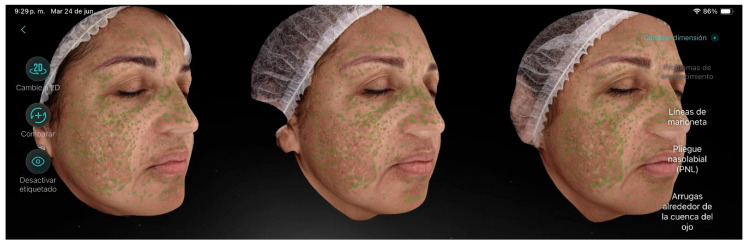
Participant 2 showing a reduction in hyperpigmentation proportion, freckles, crow’s feet, and pores.

Participant 2 (Figure 3), a 53-year-old female with a dry skin type and a moderate level of aging, presented visible wrinkles and slightly sagging skin, along with hyperpigmentation over extensive areas of the skin. The main factor contributing to aging was hyperpigmentation, as well as wrinkles around the eye contour. Additionally, she exhibited enlarged pores and low luminosity. After the treatment sessions, a reduction in the overall proportion of hyperpigmentation was observed, along with a decrease in the number of freckles, periorbital wrinkles, and pore size.

**Figure 4:**
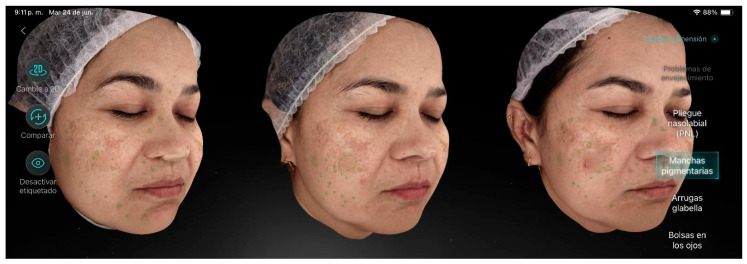
Participant 3 showing attenuation of spots (freckles and pigmentation) and pores.

Participant 3 (Figure 4), a 46-year-old female, presented in the initial skin analysis a mild level of aging with visible wrinkles even at rest, decreased skin turgor, and moderate hyperpigmentation on the eyelids and cheeks, along with pigmented dark circles. The main factors contributing to aging were hyperpigmentation and periorbital wrinkles. Additionally, she exhibited enlarged pores, blackheads, and dark circles. After completing the treatment, improvement in pigmentation was observed, along with a reduction in freckles and pore size.

**Figure 5:**
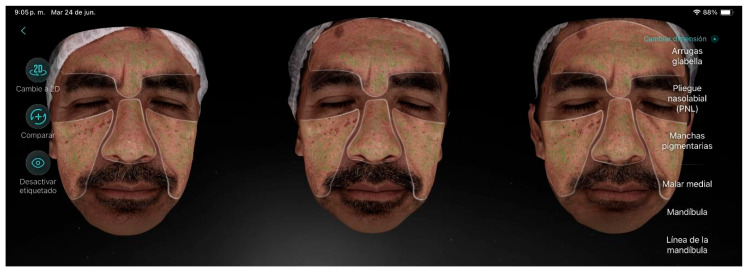
Participant 4 showing improvement in eyelid laxity, forehead lines, and marionette lines, along with reduction in pore size.

Participant 4 (Figure 5), a 51-year-old male, presented a moderate level of aging with visible wrinkles, slightly sagging skin, and hyperpigmentation over a large area of the face. The main factor contributing to aging was hyperpigmentation. He also exhibited periorbital wrinkles, enlarged pores, dull skin, and blackheads. After the treatment, no improvement was achieved in hyperpigmentation; however, there were positive results regarding skin firmness, reduction in expression lines, decreased pore size, and improved skin hydration.

**Figure 6:**
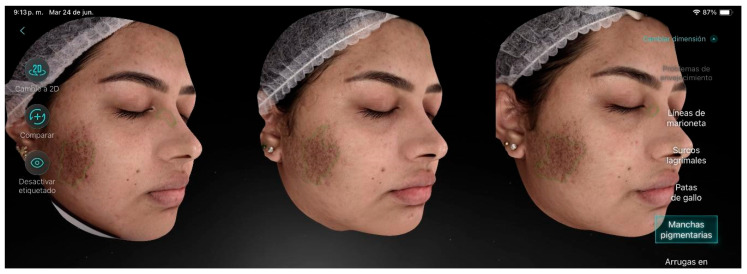
Participant 5 showing a decrease in chloasma, crow’s feet, and pores.

Participant 5 (Figure 6), a 30-year-old female, presented a mild level of aging with visibly rough skin texture and some fine lines even at rest. She also exhibited epidermal melasma with pronounced chloasma on one cheek. The main factors contributing to aging were melasma and dark circles. Additionally, she showed acne marks, under-eye bags, and enlarged pores. After the treatment sessions, positive results were observed with a reduction in chloasma, crow’s feet, and enlarged pores.

**Figure 7:**
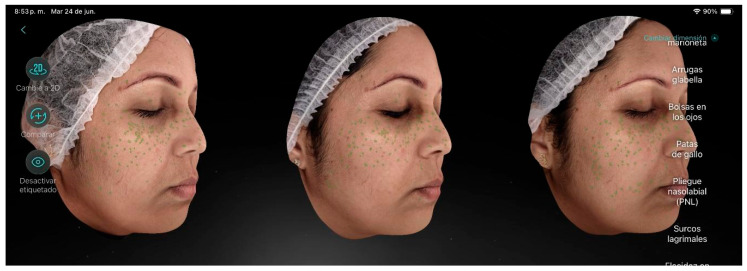
Participant 6 showing a reduction in freckles and pores.

Participant 6 (Figure 7), a 46-year-old female, presented in the initial analytical evaluation a mild degree of aging, visibly rough skin texture, slightly dehydrated skin, and some fine lines even at rest, along with dark circles, pigmented spots, freckles, crow’s feet, enlarged pores, and blackheads. After the treatment, a reduction in the number of freckles and pore size was observed, as well as increased luminosity, a more even tone, and improved skin texture.

**Figure 8:**
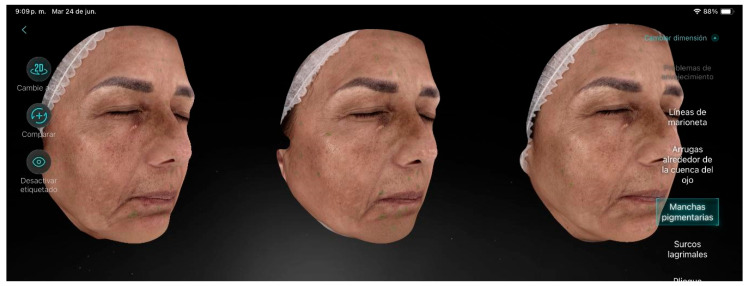
Participant 7 showing notable improvement in hyperpigmentation (chloasma, pigmentation) and pores.

Participant 7 (Figure 8), a 60-year-old female with a moderate level of aging, presented visible wrinkles and slightly sagging skin, along with severe pigmentation over extensive areas of the skin. The main factors contributing to aging were pigmented spots, crow’s feet, dark circles, and dehydrated skin. After the treatment sessions, significant improvements were observed regarding hyperpigmentation, chloasma, and overall pigmentation. Additionally, the skin showed greater luminosity, a more even tone, and a reduction in pore size.

According to the evaluated parameters, 28.6% of the participants showed improvement in hyperpigmentation proportion, 28.6% in chloasma, 57.1% in freckles, 42.9% in pigmentation, and 42.9% in crow’s feet. A reduction in pore size was observed in all participants, resulting in visibly improved skin texture.

Based on the survey conducted, participants reported satisfaction with the observed results, the treatment, the care provided by the professionals, the experience with the products both at home and in the clinic, and the at-home routine with the glutathione-based dermal serum and supplement. Participants highlighted the significant improvements in skin luminosity, expression lines, and texture, as well as an overall more even tone.

No adverse effects were reported by any participants during the intervention with the supplement, in-clinic products, or home care routines.

## 4. Discussion

The results support the hypothesis that the combination of topical and oral glutathione, within a chronocosmetic protocol, can produce significant benefits regarding clinical parameters of facial aging, particularly in relation to hyperpigmentation.

The use of glutathione as both a systemic and topical antioxidant, along with techniques that enhance skin penetration (such as microneedling), demonstrates clear synergy. This aligns with previous research showing its depigmenting and anti-wrinkle effects [4,17].

Moreover, the chronocosmetic-based application—synchronizing the times of use of active ingredients with the skin’s circadian rhythms—may have enhanced the treatment’s efficacy. This hypothesis is supported by the literature on cutaneous regeneration and defense biorhythms [18,19].

The use of glutathione as a depigmenting agent is widespread in Asian countries such as South Korea. The formulations of Korean cosmetics include ingredients that have biochemical functions in the body, which can be applied topically and supplemented orally [20]. This study confirms that this approach to functional cosmetics may be suitable to achieve beneficial results in patients with aesthetic concerns without having to resort to invasive interventions. Although it requires more time and consistent application, the absence of adverse reactions and the potential to obtain additional benefits in relation to the patients’ overall health justify its application.

It is recommended that future interventions for depigmentation combine in-clinic treatments with active antioxidant ingredients using a multidisciplinary approach. This should include not only supplementation but also dietary strategies to counteract inflammation. It is also suggested to apply this same protocol using frictionless devices (such as nanoneedles) to optimize outcomes.

For any anti-aging treatment, it is essential to consider the patient’s nutrition, as skin aging is also a consequence of poor and inadequate nutrition. To maintain healthy skin, it is necessary to ensure sufficient water intake; reduce the consumption of fats, refined flours, and sugars; and increase the intake of foods rich in antioxidants (fruits, vegetables, whole grains, nuts, seeds, and spices), collagen peptides (lean meats, fish, bone broth, gelatin, dairy products, eggs, citrus fruits, and berries), polyphenols (tea polyphenols, curcumin, flavonoids, silymarin, and grape-derived resveratrol), polysaccharides (such as agaric polysaccharides, Lycium polysaccharides, algae polysaccharides, lingzhi polysaccharides, and mushroom-derived polysaccharides), vitamins (A, B complex (B3, B12), E, D, C, coenzyme Q10, and lipoic acid), fatty acids (omega-3 and omega-6, oral olive oil), and other nutrients (dietary probiotics); and eliminate tobacco and alcohol consumption [21].

Although the results are promising, the small sample size limits the generalizability of the findings. Nevertheless, the data obtained can provide a foundation for future studies involving control groups and long-term follow-up.

### Limitations

Although this study provides relevant findings on the effects of glutathione on visible signs of facial aging within a chronocosmetic framework, it has several limitations that must be considered when interpreting the results:

**Small sample size (*n* = 7)**: The limited number of participants restricts the generalizability of the findings, and the lack of statistical power prevents causal inference. While visible improvements were observed across multiple parameters (radiance, expression lines, and pigmentation), these results cannot be statistically extrapolated to a broader population and must be verified with larger, controlled studies.

**Lack of a control or placebo group**: As this was an interventional study without a comparison group, the observed changes cannot be definitively attributed to the glutathione protocol. Other factors, such as the placebo effect, adherence to a consistent skincare routine, or individual variability in circadian rhythms, may have influenced the results.

**Study duration**: The intervention lasted 7 weeks, which may be insufficient to capture the full effects of deep dermal regeneration processes or to assess the long-term sustainability of the results. Studies with 3- or 6-month follow-ups could yield more conclusive data on treatment durability.

**Individual variability**: Although Fitzpatrick skin types III and IV were included, other variables, such as diet, stress levels, sleep quality, and daily sun exposure, were not controlled. These factors could interfere with skin response and circadian rhythm regulation.

**Standardization of the home-care protocol**: Despite clear instructions being provided, there was no daily compliance monitoring system for the participants. This may have affected the consistency of the protocol and, consequently, the interpretation of the results.

**Access to the complete data**: The results shown in Table 1 are a synthesis of the data from each patient provided by the camera. For research or academic purposes, detailed reports can be sent by the corresponding author upon request.

## 5. Conclusions

The combined intervention with topical and oral glutathione, which was applied in line with chronocosmetic principles, showed promising effects in improving signs of facial aging, particularly hyperpigmentation parameters, expression lines in the periorbital area, and pore size.

The application of the serum according to circadian cycles (day and night) appears to have contributed to the protocol’s effectiveness.

Microneedling with nanoneedles was found to be an effective and safe delivery method.

No adverse effects were reported during the study, and participant satisfaction was high, with noticeable improvements in skin texture, expression lines, tone, and radiance.

It is recommended that these findings be validated in controlled studies with larger sample sizes to establish clinical guidelines for the use of glutathione with a chronocosmetic approach in aesthetic medicine.

## Figures and Tables

**Table 1:** Results of baseline analytical and 3D photographic evaluation.

Participants	Initial Session						Session 4						Session 7					
	Signs of Aging					Skin Conditions	Signs of Aging					Skin Conditions	Signs of Aging					Skin Conditions
	Pigmentation Spots				Dynamic Wrinkles	Skin Conditions	Pigmentation Spots				Dynamic Wrinkles	Skin Conditions	Pigmentation Spots				Dynamic Wrinkles	Skin Conditions
	Proportion	Chloasma	Freckles	Pigmentation	Crow’s Feet	Pores	Proportion	Chloasma	Freckles	Pigmentation	Crow’s Feet	Pores	Proportion	Chloasma	Freckles	Pigmentation	Crow’s Feet	Pores
**P 1**	12.08%	8	386	98	2.5	62	10.92%	9	378	100	2.1	52	11.41%	10	330	71	2.3	43
**P 2**	34.62%	11	732	49	4.6	62	33.19%	13	633	79	3.4	60	32.75%	12	691	56	3.8	50
**P 3**	11.80%	6	367	51	5.1	71	14.10%	8	379	51	4.9	69	12.04%	7	335	37	5.7	62
**P 4**	18.99%	13	592	20	6.5	38	14.77%	10	471	39	6.5	32	22.87%	13	665	32	6.6	27
**P 5**	4.71%	8	82	23	2.2	73	5.59%	5	114	31	2.2	64	4.81%	5	126	38	2.1	56
**P 6**	4.11%	6	237	34	4.4	91	4.51%	7	200	35	4.3	87	6.70%	7	229	39	4.5	77
**P 7**	15.57%	17	377	26	4.8	57	22.28%	14	416	36	5.1	49	21.33%	15	436	24	5.3	49

## Data Availability

The data supporting the findings of this study can be made available upon request.

## Supplementary Materials

The following supporting information can be downloaded at: https://www.mdpi.com/article/10.3390/life15101623/s1, Figure S1: Pigmented spots (proportion)—3D imaging; Figure S2: Chloasma—3D imaging; Figure S3: Freckles—3D imaging; Figure S4: Pigmentation—3D imaging; Figure S5: Crow’s feet—3D imaging; Figure S6: Pores—3D imaging.
