# Supplementary material for: A Chronocosmetic Approach to Treating Signs of Aging with Glutathione
Source: Life (Basel). 2025 Oct 17;15(10):1623. doi: 10.3390/life15101623 (PMC12564977; doi:10.3390/life15101623)
Supplement: Supplementary file 1 [file life-15-01623-s001.zip › life-3781248-Supplementary.pdf]

Supplementary Materials

The following indicators were measured to assess the progression of various skin aging parameters: hyperpigmentation (proportion, chloasma, freckles, and pigmentation), expression lines (crow's Feet), and skin issues (pores).

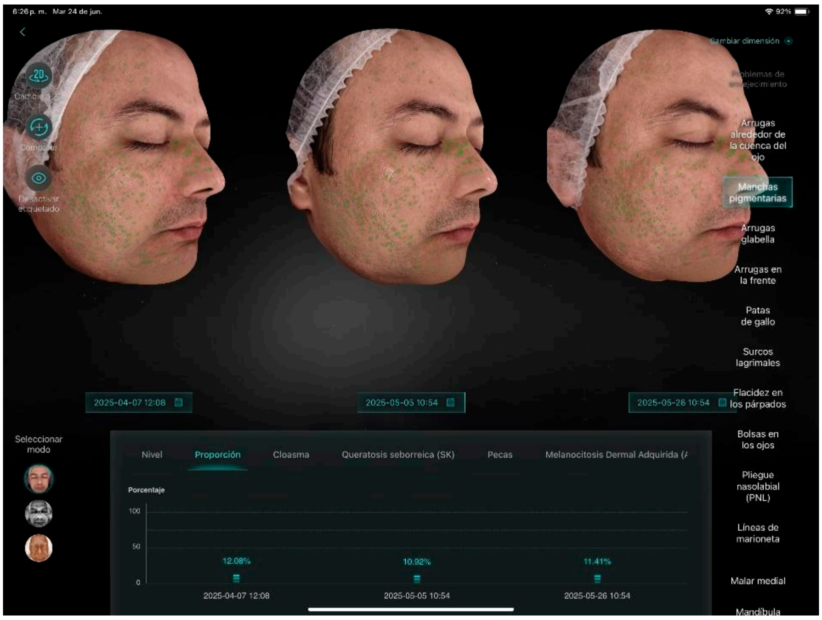

Figure S1. Pigmented spots (proportion)—3D imaging.

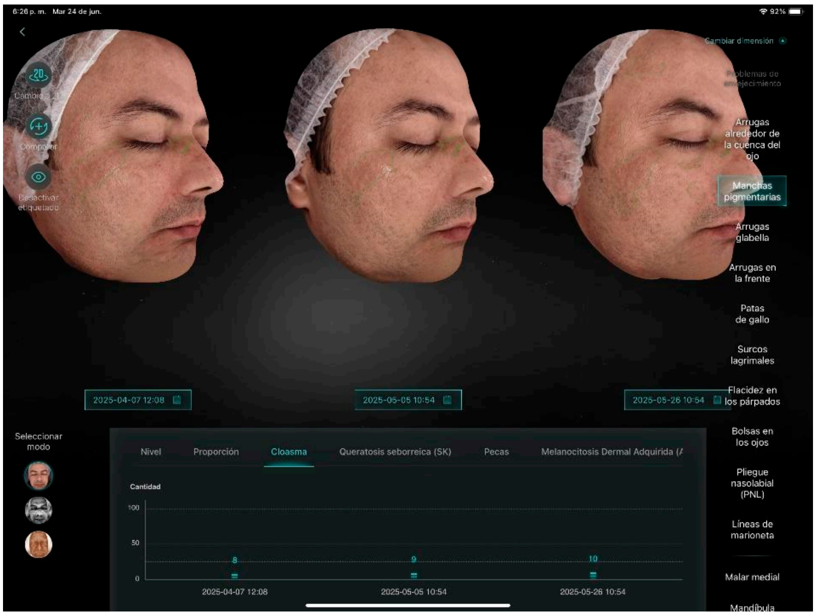

Figure S2. Chloasma—3D imaging.

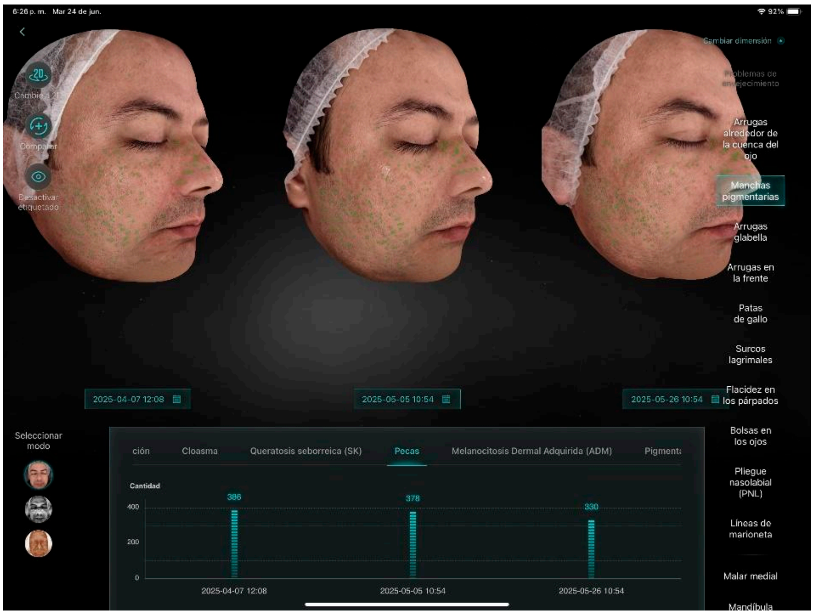

Figure S3. Freckles—3D imaging.

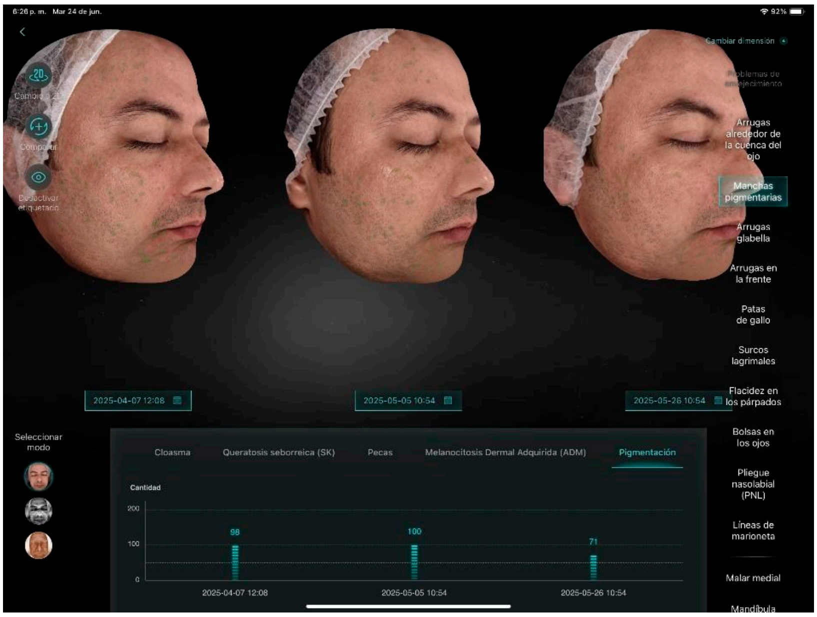

Figure S4. Pigmentation—3D imaging.

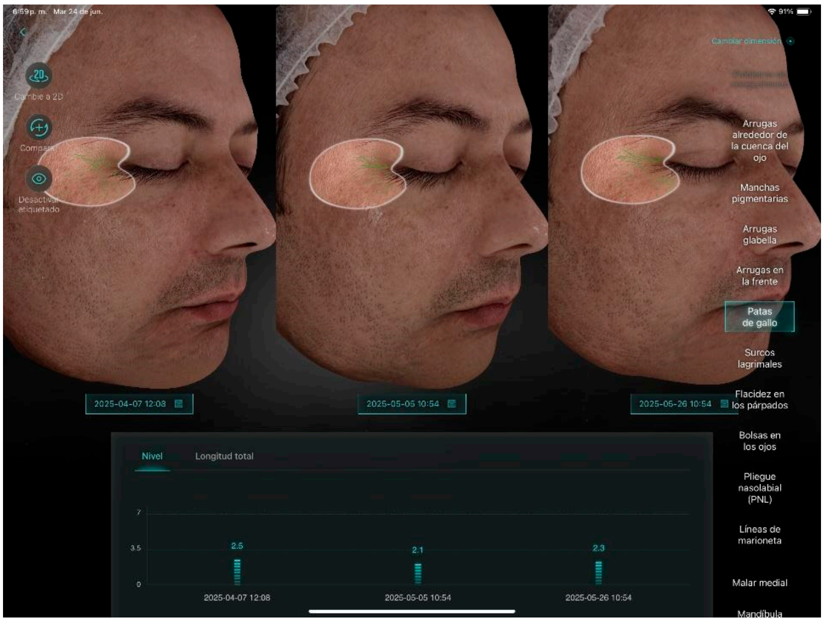

Figure S5. Crow's feet—3D imaging.

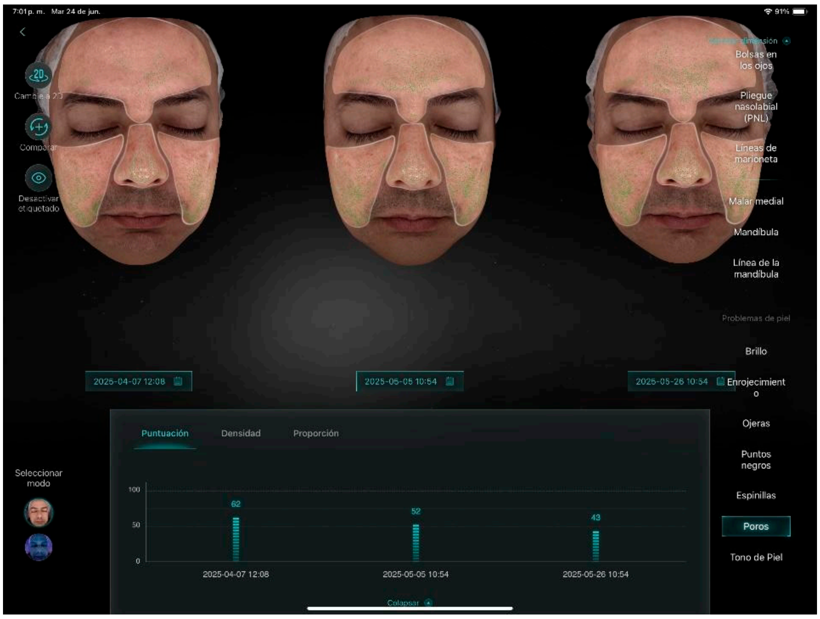

Figure S6. Pores—3D imaging.

The intervention phase was carried out from weeks 2 to 8, during which in-clinic protocols were performed using nanoneedles to apply the dose of glutathione. Participants were instructed to follow a special home care protocol that included topical glutathione application (morning and night) and daily oral administration. After completing the first 5 weeks of treatment, a follow-up phase was conducted, including photographic documentation with standard photos and a 3D camera. At the end of week 8, a cosmetic re-

evaluation was performed, along with photographic and 3D imaging, and an assessment of patient satisfaction and monitoring of potential adverse effects.
